# Crystal structure of NH_4_[La(SO_4_)_2_(H_2_O)]

**DOI:** 10.1107/S2056989015009457

**Published:** 2015-05-23

**Authors:** Meriem Benslimane, Yasmine Kheira Redjel, Hocine Merazig, Jean-Claude Daran

**Affiliations:** a Unité de Recherche de Chimie de l’Environnement et Moléculaire Structurale, Faculté des Sciences Exactes, Département de Chimie, Université de Constantine 1, 25000 Constantine, Algeria; b Laboratoire de Chimie de Coordination, UPR-CNRS 8241, 05 route de Narbonne, 31077 Toulouse Cedex 4

**Keywords:** crystal structure, hydrous ternary sulfates, hydro­thermal synthesis, hydrogen bonding

## Abstract

The principal building units in the crystal structure of ammonium aqua­bis(sulfato)­lanthanate(III) are slightly distorted SO_4_ tetra­hedra, LaO_9_ polyhedra in the form of distorted tricapped trigonal prisms, and NH_4_^+^ ions. The La^3+^ cation is coordinated by eight O atoms from six different sulfate tetra­hedra, two of which are bidentate coordinating and four monodentate, as well as one O atom from a water mol­ecule; each sulfate anion bridges three La^3+^ cations. These bridging modes result in the formation of a three-dimensional anionic [La(SO_4_)_2_(H_2_O)]^−^ framework that is stabilized by O—H⋯O hydrogen-bonding inter­actions. The disordered ammonium cations are situated in the cavities of this framework and are hydrogen-bonded to six surrounding O atoms.

## Abstract 2

The structure of (NH_4_)[La(SO_4_)_2_(H_2_O)] comprises LaO_9_ polyhedra and SO_4_ tetra­hedra, which are linked by common edges and vertices, forming a three-dimensional network with the hydrogen-bonded NH_4_^+^ ions in the cavities.

## Chemical context   

Three-dimensional framework materials are characterized by their structural diversity. They are of continuing inter­est as a result of their technologically important properties and potential applications in catalysis, ion-exchange, adsorption, inter­calation, and radioactive waste remediation (Szostak, 1989 ▸; Cheetham *et al.*, 1999 ▸; Rosi *et al.*, 2003 ▸; Ok *et al.*, 2007 ▸). Many materials showing such functional features contain structurally versatile cations, in particular heavier metal cations with large coordination spheres. Among many other cations, lanthanide cations have been used widely, since they exhibit high coordination numbers and can show a large topological diversity in the resulting framework structures (Bataille & Louër, 2002 ▸; Wickleder, 2002 ▸; Yuan *et al.*, 2005 ▸). One of the most promising synthetic methods for the preparation of compounds with framework structures is the hydro­thermal (or solvothermal) reaction technique (Feng *et al.*, 1997 ▸; Natarajan *et al.*, 2000 ▸) in which mineralizers such as acids or bases are introduced to increase the solubility and reactivity of the reagents (Laudise, 1959 ▸; Laudise & Ballman, 1958 ▸). Moreover, organic or inorganic templates are used to direct the topologies of the framework structures and the concomitant physical and chemical properties of the products (Szostak, 1989 ▸; Breck, 1974 ▸; Barrer, 1982 ▸). Thus, we have tried to utilize the hydro­thermal technique to react a lanthanide cation (La^3+^) with sulfuric acid in the presence of NH_4_OH and 3-amino­benzoic acid as a template to prepare higher dimensional framework materials. However, in the present case the organic template was not incorporated in the resultant crystal structure of the title compound, NH_4_[La(SO_4_)_2_(H_2_O)], which represents a new hydrate form. Other members in the system NH_4_^+^/La^3+^/SO_4_^2−^/(H_2_O) are two forms of anhydrous (NH_4_)[La(SO_4_)_2_] (Sarukhanyan *et al.*, 1984*a* ▸; Bénard-Rocherullé *et al.*, 2001 ▸), (NH_4_)_5_[La(SO_4_)_4_] (Niinisto *et al.*, 1980 ▸) and (NH_4_)[La(SO_4_)_2_(H_2_O)_4_] (Keppert *et al.*, 1999 ▸).

Sulfates with an *A* ^+^:*Ln* ^3+^ (*A* ^+^ = alkaline ions, *Ln* ^3+^ = lanthanide ions) ratio of 1:1 are one of the best investigated groups among hydrous ternary sulfates. They crystallize either as monohydrates (Blackburn & Gerkin, 1995 ▸; Barnes, 1995 ▸; Iskhakova *et al.*, 1985*a* ▸) or tetra­hydrates (Eriksson *et al.*, 1974 ▸), and in few cases also as dihydrates (Kaucic *et al.*, 1985 ▸; Iskhakova & Trunov, 1985 ▸). The tetra­hydrates are mainly found for the bigger monovalent ions Cs^+^, NH_4_^+^, and Rb^+^. For the smaller *A* ^+^ ions such as Na^+^, the monohydrate becomes dominant.

## Structural commentary   

**Figure 1:**
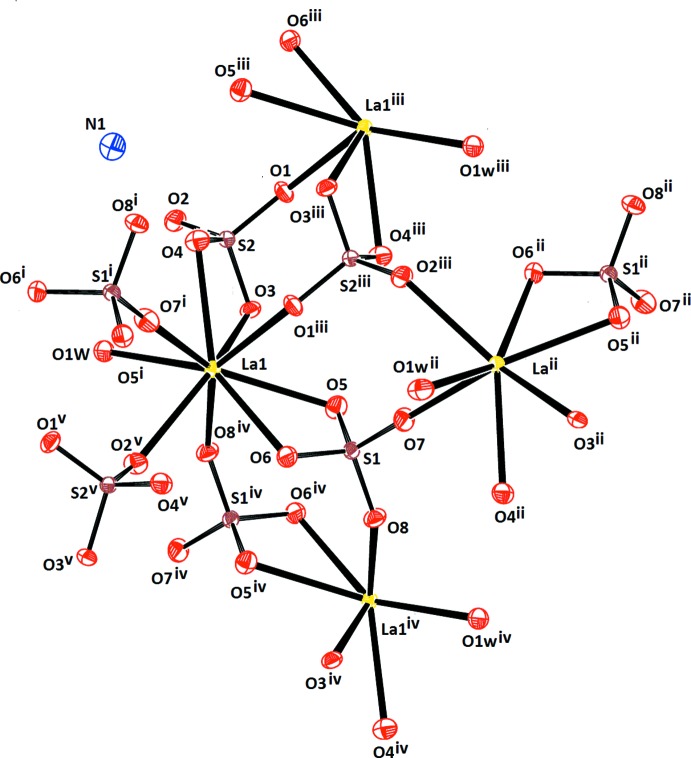
The principal building units, LaO_9_ polyhedra and SO_4_ tetra­hedra, in the crystal structure of (NH_4_)[La(SO_4_)_2_(H_2_O)], showing the atom-labelling scheme. Displacement ellipsoids are drawn at the 50% probability level. [Symmetry codes: (i) 1 − *x*, −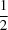 + *y*, 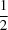 − *z*; (ii) 1 − *x*, 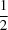 + *y*, 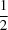 − *z*; (iii) 1 − *x*, 2 − *y*, −*z*; (iv) 2 − *x*, 2 − *y*, 1 − *z*; (v) *x*, 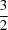 − *y*, 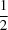 + *z*.]

**Figure 2:**
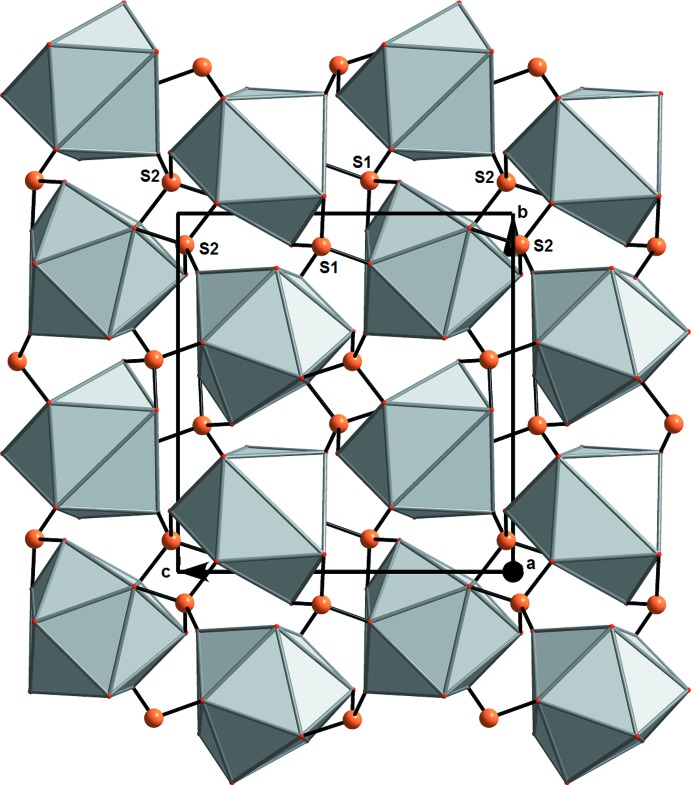
The connection of LaO_9_ polyhedra and SO_4_ tetra­hedra in the crystal structure of (NH_4_)[La(SO_4_)_2_(H_2_O)], viewed along the *a* axis.

The structure of the title compound comprises LaO_9_ polyhedra and SO_4_ tetra­hedra as the principal building units (Fig. 1 ▸), forming an anionic [La(SO_4_)_2_(H_2_O)]^−^ framework by sharing common edges and vertices (Fig. 2 ▸). The NH_4_^+^ counter-cations are situated in the cavities of this framework.

The La^3+^ cation is coordinated by eight O atoms from six different sulfate tetra­hedra. Two tetra­hedra are in a bidentate coordination mode and four tetra­hedra are in a monodentate mode. The distorted tricapped trigonal–prismatic coordination sphere is completed by one O atom from a water mol­ecule. The La—O bond lengths, ranging from 2.472 (3) to 2.637 (3) Å with 2.496 (3) Å to the water mol­ecule, and the O—La—O angles, ranging from 53.55 (8) to 145.43 (9)°, are similar to the analogous distances found in NaLa(SO_4_)_2_·H_2_O (Blackburn & Gerkin, 1995 ▸). The ninefold coordination of La^3+^ in NH_4_[La(SO_4_)_2_(H_2_O)] is typical for the majority of monohydrated alkali rare earth sulfate complexes and of rare earth complexes in general. For early members of the rare earth sulfate series, the coordination number of nine is realized, *e.g.* for Ce, Pr, La and Nd (Blackburn & Gerkin, 1994 ▸, 1995 ▸; Iskhakova *et al.*, 1985*b* ▸, 1988 ▸). For later members of the sulfate series, such as Gd (Sarukhanyan *et al.*, 1984*b* ▸), the coordination number decreases to eight, presumably in association with the lanthanide contraction. There are two sulfur atoms (S1, S2) in the asymmetric unit of the title compound, both with very similar S—O bond lengths in the ranges 1.465 (3)–1.488 (3) and 1.468 (3)–1.490 (3) Å, respectively. The range of O—S—O bond angles, 106.04 (16)–110.89 (19)° for S1 and 104.70 (16)–111.52 (17)° for S2, reflect the distortion of the two sulfate tetra­hedra. Each SO_4_ anion bridges three La^3+^ cations (Fig. 2 ▸).

## Supra­molecular features   

The bridging modes of the O atoms result in the formation of a three-dimensional anionic framework, stabilized by O—H⋯O hydrogen-bonding inter­actions between the aqua ligand and the two SO_4_ tetra­hedra (Table 1 ▸) whereby each sulfate tetra­hedron establishes one hydrogen bond with the water mol­ecule *via* the oxygen atom (O6 and O3) corres­ponding to the longest S—O bonds. The N atoms are situated in the cavities of this framework. Although the H atoms of the ammonium cation could not be located, the N⋯O distances between 2.865 (5) and 3.036 (5) Å strongly suggest N—H⋯O hydrogen bonds of medium strength (Table 1 ▸). It appears most likely that the number of O atoms (six) in the vicinity of the N atom is the reason for the disorder of the ammonium cation.

## Synthesis and crystallization   

The title compound was obtained during the attempted preparation of a complex resulting from the hydro­thermal reaction of La_2_O_3_ (0.1 g, 1 mmol) with 37%wt sulfuric acid and 3-amino­benzoic acid (0.048 g, 1 mmol) in the presence of NH_4_OH in 10 ml water. The mixture was kept in a 23 ml Teflon-lined steel autoclave at 433 K for 3 d. After this treatment, the autoclave was cooled slowly to room temperature. Slow evaporation of the solvent at room temperature led to the formation of prismatic colourless crystals of the title compound.

## Refinement   

Crystal data, data collection and structure refinement details are summarized in Table 2 ▸. The oxygen-bound hydrogen atoms were located in a difference Fourier map and were refined with restraints of the O—H bond length [0.85 (1) Å] and H⋯H distances (1.39 Å) and with *U*_iso_(H) = 1.5*U*_eq_(O). The ammonium hydrogen atoms could not be located reliably by difference Fourier methods. Several disorder models considering the hydrogen-bonding environment (see Table 1 ▸) failed, eventually leading to the exclusion of the ammonium hydrogen atoms from the refinement. The maximum and minimum peaks in the final difference Fourier map are 0.93 and 0.72 Å, respectively, from atom La1.

Diffraction data were collected some time ago, and merged in the corresponding crystal class. Unfortunately, the original measurement data got lost; experiments to repeat the crystal growth were unsuccessful. Therefore the crystal structure was finally solved and refined with the merged data set.

## Supplementary Material

Crystal structure: contains datablock(s) I, global. DOI: 10.1107/S2056989015009457/wm5148sup1.cif

Structure factors: contains datablock(s) I. DOI: 10.1107/S2056989015009457/wm5148Isup2.hkl

CCDC reference: 1401662

Additional supporting information:  crystallographic information; 3D view; checkCIF report

## Figures and Tables

**Table 1:** Hydrogen-bond geometry (, )

*D*H*A*	*D*H	H*A*	*D* *A*	*D*H*A*
O1*W*H11O3^i^	0.84(5)	1.94(5)	2.717(5)	153(5)
O1*W*H21O6^ii^	0.85(3)	1.95(3)	2.778(4)	168(5)
N1O1^iii^			2.942(5)	
N1O6^ii^			3.036(5)	
N1O3^iv^			2.914(5)	
N1O8^v^			2.943(5)	
N1O5^vi^			2.865(5)	
N1O4			2.866(5)	

Symmetry codes: (i) 
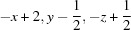
; (ii) 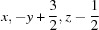; (iii) 
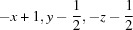
; (iv) 
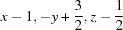
; (v) 
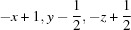
; (vi) 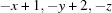.

**Table 2:** Experimental details

Crystal data	
Chemical formula	NH_4_[La(SO_4_)_2_(H_2_O)]
*M*_r_	367.07
Crystal system, space group	Monoclinic, *P*2_1_/*c*
Temperature (K)	100
*a*, *b*, *c* ()	8.4919(16), 9.978(2), 11.9268(19)
()	128.511(10)
*V* (^3^)	790.7(3)
*Z*	4
Radiation type	Mo *K*
(mm^1^)	5.96
Crystal size (mm)	0.30 0.20 0.10

Data collection	
Diffractometer	Nonius KappaCCD
Absorption correction	For a sphere (Dwiggins, 1975 ▸)
*T*_min_, *T*_max_	0.419, 0.431
No. of measured, independent and observed [*I* > 2(*I*)] reflections	2414, 2414, 2362
(sin /)_max_ (^1^)	0.715

Refinement	
*R*[*F* ^2^ > 2(*F* ^2^)], *wR*(*F* ^2^), *S*	0.027, 0.081, 1.26
No. of reflections	2414
No. of parameters	124
No. of restraints	3
H-atom treatment	H atoms treated by a mixture of independent and constrained refinement
_max_, _min_ (e ^3^)	1.81, 1.48

Computer programs: *COLLECT* (Nonius, 1998 ▸), *DENZO* and *SCALEPACK* (Otwinowski Minor, 1997 ▸), *SIR92* (Altomare *et al.*, 1993 ▸), *SHELXL97* (Sheldrick, 2008 ▸), *ORTEP-3 for Windows* and *WinGX* (Farrugia, 2012 ▸) and *DIAMOND* (Brandenburg Berndt, 1999 ▸).

## supplementary crystallographic information

### Crystal data

**Table:**

NH_4_[La(SO_4_)_2_(H_2_O)]	*F*(000) = 680
*M**_r_* = 367.07	*D*_x_ = 3.083 Mg m^−^^3^
Monoclinic, *P*2_1_/*c*	Mo *K*α radiation, λ = 0.71073 Å
Hall symbol: -P 2ybc	Cell parameters from 2542 reflections
*a* = 8.4919 (16) Å	θ = 3–30.5°
*b* = 9.978 (2) Å	µ = 5.96 mm^−^^1^
*c* = 11.9268 (19) Å	*T* = 100 K
β = 128.511 (10)°	Prism, colourless
*V* = 790.7 (3) Å^3^	0.30 × 0.20 × 0.10 × 0.10 (radius) mm
*Z* = 4	

### Data collection

**Table:**

Nonius KappaCCD diffractometer	2414 independent reflections
Radiation source: fine-focus sealed tube	2362 reflections with *I* > 2σ(*I*)
Graphite monochromator	*R*_int_ = 0.0000
Detector resolution: 9 pixels mm^-1^	θ_max_ = 30.5°, θ_min_ = 3.0°
CCD scans	*h* = −12→0
Absorption correction: for a sphere (Dwiggins, 1975)	*k* = −14→0
*T*_min_ = 0.419, *T*_max_ = 0.431	*l* = −12→17
2414 measured reflections	

### Refinement

**Table:**

Refinement on *F*^2^	Primary atom site location: structure-invariant direct methods
Least-squares matrix: full	Secondary atom site location: difference Fourier map
*R*[*F*^2^ > 2σ(*F*^2^)] = 0.027	Hydrogen site location: inferred from neighbouring sites
*wR*(*F*^2^) = 0.081	H atoms treated by a mixture of independent and constrained refinement
*S* = 1.26	*w* = 1/[σ^2^(*F*_o_^2^) + (0.0427*P*)^2^ + 2.7376*P*] where *P* = (*F*_o_^2^ + 2*F*_c_^2^)/3
2414 reflections	(Δ/σ)_max_ = 0.001
124 parameters	Δρ_max_ = 1.81 e Å^−^^3^
3 restraints	Δρ_min_ = −1.48 e Å^−^^3^

### Special details

**Table:**

Geometry. All e.s.d.'s (except the e.s.d. in the dihedral angle between two l.s. planes) are estimated using the full covariance matrix. The cell e.s.d.'s are taken into account individually in the estimation of e.s.d.'s in distances, angles and torsion angles; correlations between e.s.d.'s in cell parameters are only used when they are defined by crystal symmetry. An approximate (isotropic) treatment of cell e.s.d.'s is used for estimating e.s.d.'s involving l.s. planes.
Refinement. Refinement of *F*^2^ against ALL reflections. The weighted *R*-factor *wR* and goodness of fit *S* are based on *F*^2^, conventional *R*-factors *R* are based on *F*, with *F* set to zero for negative *F*^2^. The threshold expression of *F*^2^ > σ(*F*^2^) is used only for calculating *R*-factors(gt) *etc*. and is not relevant to the choice of reflections for refinement. *R*-factors based on *F*^2^ are statistically about twice as large as those based on *F*, and *R*- factors based on ALL data will be even larger.

### Fractional atomic coordinates and isotropic or equivalent isotropic displacement parameters (Å^2^)

**Table:**

	*x*	*y*	*z*	*U*_iso_*/*U*_eq_	
La1	0.71683 (3)	0.839390 (18)	0.248314 (19)	0.01052 (8)	
S1	0.74128 (12)	1.09162 (8)	0.42791 (8)	0.01163 (15)	
S2	0.70608 (12)	0.91270 (8)	−0.02026 (8)	0.01129 (15)	
O1	0.6085 (4)	1.0290 (3)	−0.1156 (3)	0.0179 (5)	
O2	0.8105 (5)	0.8337 (3)	−0.0602 (3)	0.0189 (5)	
O3	0.8535 (4)	0.9585 (3)	0.1310 (3)	0.0156 (5)	
O8	0.9057 (4)	1.1402 (3)	0.5727 (3)	0.0182 (5)	
O4	0.5597 (4)	0.8301 (3)	−0.0221 (3)	0.0188 (5)	
O7	0.5667 (4)	1.1797 (3)	0.3641 (3)	0.0229 (6)	
O6	0.6873 (4)	0.9516 (3)	0.4347 (3)	0.0185 (5)	
O5	0.8062 (4)	1.0870 (3)	0.3387 (3)	0.0192 (5)	
O1W	0.8711 (5)	0.6537 (3)	0.2059 (3)	0.0241 (6)	
H11	0.982 (5)	0.615 (6)	0.266 (4)	0.036*	
H21	0.820 (8)	0.632 (6)	0.121 (2)	0.036*	
N1	0.2567 (6)	0.6458 (4)	−0.2302 (4)	0.0244 (7)	

### Atomic displacement parameters (Å^2^)

**Table:**

	*U*^11^	*U*^22^	*U*^33^	*U*^12^	*U*^13^	*U*^23^
La1	0.01098 (11)	0.00981 (11)	0.01113 (11)	−0.00037 (5)	0.00706 (9)	−0.00028 (5)
S1	0.0106 (3)	0.0110 (3)	0.0114 (3)	0.0009 (3)	0.0059 (3)	−0.0005 (3)
S2	0.0118 (3)	0.0111 (3)	0.0101 (3)	0.0008 (3)	0.0064 (3)	0.0000 (2)
O1	0.0212 (12)	0.0144 (11)	0.0173 (11)	0.0055 (10)	0.0116 (10)	0.0056 (9)
O2	0.0214 (13)	0.0207 (14)	0.0180 (13)	0.0035 (10)	0.0140 (12)	−0.0013 (9)
O3	0.0158 (11)	0.0162 (11)	0.0111 (10)	−0.0037 (9)	0.0066 (9)	−0.0028 (9)
O8	0.0141 (12)	0.0206 (12)	0.0141 (12)	−0.0009 (10)	0.0059 (10)	−0.0041 (10)
O4	0.0181 (13)	0.0212 (13)	0.0174 (12)	−0.0061 (9)	0.0111 (11)	−0.0018 (9)
O7	0.0139 (12)	0.0207 (13)	0.0217 (13)	0.0068 (10)	0.0049 (11)	−0.0025 (10)
O6	0.0240 (13)	0.0145 (11)	0.0174 (12)	−0.0029 (10)	0.0130 (11)	−0.0002 (9)
O5	0.0246 (13)	0.0181 (12)	0.0211 (12)	−0.0020 (10)	0.0174 (11)	−0.0016 (10)
O1W	0.0289 (16)	0.0220 (14)	0.0177 (13)	0.0126 (11)	0.0127 (12)	0.0010 (10)
N1	0.0248 (16)	0.0252 (18)	0.0296 (18)	−0.0037 (13)	0.0201 (15)	−0.0006 (13)

### Geometric parameters (Å, º)

**Table:**

La1—O7^i^	2.472 (3)	S1—O8	1.471 (3)
La1—O1W	2.496 (3)	S1—O5	1.472 (3)
La1—O8^ii^	2.521 (3)	S1—O6	1.488 (3)
La1—O1^iii^	2.533 (3)	S2—O1	1.468 (3)
La1—O2^iv^	2.563 (3)	S2—O2	1.470 (3)
La1—O3	2.596 (3)	S2—O4	1.480 (3)
La1—O5	2.612 (3)	S2—O3	1.490 (3)
La1—O4	2.614 (3)	O1W—H11	0.845 (10)
La1—O6	2.637 (3)	O1W—H21	0.844 (10)
S1—O7	1.465 (3)		
			
O7^i^—La1—O1W	82.44 (12)	O7^i^—La1—O6	99.16 (10)
O7^i^—La1—O8^ii^	143.78 (10)	O1W—La1—O6	145.43 (9)
O1W—La1—O8^ii^	71.36 (10)	O8^ii^—La1—O6	89.43 (9)
O7^i^—La1—O1^iii^	71.36 (10)	O1^iii^—La1—O6	70.57 (9)
O1W—La1—O1^iii^	139.83 (10)	O2^iv^—La1—O6	71.00 (9)
O8^ii^—La1—O1^iii^	143.55 (9)	O3—La1—O6	124.69 (8)
O7^i^—La1—O2^iv^	72.90 (10)	O5—La1—O6	53.55 (8)
O1W—La1—O2^iv^	76.67 (10)	O4—La1—O6	144.28 (9)
O8^ii^—La1—O2^iv^	76.96 (10)	O7—S1—O8	109.04 (17)
O1^iii^—La1—O2^iv^	121.24 (9)	O7—S1—O5	110.89 (19)
O7^i^—La1—O3	127.89 (10)	O8—S1—O5	110.49 (17)
O1W—La1—O3	76.32 (10)	O7—S1—O6	110.19 (18)
O8^ii^—La1—O3	70.11 (9)	O8—S1—O6	110.17 (16)
O1^iii^—La1—O3	96.07 (9)	O5—S1—O6	106.04 (16)
O2^iv^—La1—O3	142.55 (9)	O7—S1—La1	119.80 (13)
O7^i^—La1—O5	140.16 (10)	O8—S1—La1	131.15 (12)
O1W—La1—O5	137.02 (11)	O5—S1—La1	52.71 (11)
O8^ii^—La1—O5	71.62 (9)	O6—S1—La1	53.78 (11)
O1^iii^—La1—O5	72.00 (9)	O1—S2—O2	109.67 (16)
O2^iv^—La1—O5	114.84 (9)	O1—S2—O4	111.40 (17)
O3—La1—O5	71.17 (8)	O2—S2—O4	111.52 (17)
O7^i^—La1—O4	74.43 (10)	O1—S2—O3	109.85 (16)
O1W—La1—O4	69.69 (10)	O2—S2—O3	109.59 (17)
O8^ii^—La1—O4	116.81 (9)	O4—S2—O3	104.70 (16)
O1^iii^—La1—O4	74.17 (9)	La1—O1W—H11	128 (4)
O2^iv^—La1—O4	135.44 (9)	La1—O1W—H21	119 (4)
O3—La1—O4	53.65 (8)	H11—O1W—H21	112 (3)
O5—La1—O4	109.69 (9)		

Symmetry codes: (i) −*x*+1, *y*−1/2, −*z*+1/2; (ii) −*x*+2, −*y*+2, −*z*+1; (iii) −*x*+1, −*y*+2, −*z*; (iv) *x*, −*y*+3/2, *z*+1/2.

### Hydrogen-bond geometry (Å, º)

**Table:**

*D*—H···*A*	*D*—H	H···*A*	*D*···*A*	*D*—H···*A*
O1*W*—H11···O3^v^	0.84 (5)	1.94 (5)	2.717 (5)	153 (5)
O1*W*—H21···O6^vi^	0.85 (3)	1.95 (3)	2.778 (4)	168 (5)
N1···O1^vii^			2.942 (5)	
N1···O6^vi^			3.036 (5)	
N1···O3^viii^			2.914 (5)	
N1···O8^i^			2.943 (5)	
N1···O5^iii^			2.865 (5)	
N1···O4			2.866 (5)	

Symmetry codes: (i) −*x*+1, *y*−1/2, −*z*+1/2; (iii) −*x*+1, −*y*+2, −*z*; (v) −*x*+2, *y*−1/2, −*z*+1/2; (vi) *x*, −*y*+3/2, *z*−1/2; (vii) −*x*+1, *y*−1/2, −*z*−1/2; (viii) *x*−1, −*y*+3/2, *z*−1/2.
